# Lifestraw Family water filters in low- and middle-income countries: a systematic review and meta-analysis to define longer-term public health impact against childhood diarrhoea and inform scale-up

**DOI:** 10.7189/jogh.14.04018

**Published:** 2024-06-28

**Authors:** Melissa Kerr, Vincenzo Cardinale, Corrado De Vito, Amal R Khanolkar

**Affiliations:** 1 Department of Population Health Sciences, King’s College London, Guy’s Campus, London, England, UK; 2 Department of Public Health and Infectious Diseases, Sapienza University of Rome, Rome, Italy; 3 Department of Medico-Surgical Sciences and Biotechnologies, Sapienza University of Rome, Latina, Italy

## Abstract

**Background:**

Diarrhoeal disease disproportionately affects children <5 years in low- and middle-income countries (LMICs). The pathogens responsible for diarrhoea are commonly transmitted through faecally-contaminated drinking water. Lifestraw Family point-of-use water filters have been the subject of intervention studies for over a decade and were the first filters evaluated by the World Health Organization in its water treatment evaluation scheme to provide comprehensive protection against many diarrhoea-causing pathogens. This systematic review aimed to: 1) report on aspects related to physical environment and implementation and 2) conduct an updated meta-analysis on Lifestraw Family filter effectiveness against childhood diarrhoea based on studies with ≥12 months of follow-up.

**Methods:**

We conducted a literature search in November 2022 using MEDLINE, Embase, Cochrane, and CINAHL databases. Inclusion criteria were: 1) RCTs, cluster-RCTs, quasi-experimental, or matched cohort studies on 2) Lifestraw Family 1.0 or 2.0 filters 3) conducted in LMICs 4) that evaluated filter effectiveness against diarrhoea in children <5 and 5) analysed ≥12 months of follow-up data on clinical effectiveness against diarrhoea and were 6) published from 2010 with 7) full-text availability in English. A modified Newcastle-Ottawa Scale was used to assess risk of bias. Relative risk (RR) and 95% confidence intervals (CIs) were extracted and analysed using a random-effects meta-analysis.

**Results:**

We included 6 studies in LMICs involving 4740 children <5. Of the four clinically-effective interventions, common characteristics were access to improved water sources (75%), the 2.0 version of the filter or the 1.0 version with additional water storage (100%), use of behaviour change theory, community engagement, and health messaging (75%), local filter repair-and-replace mechanisms (75%), and specially-trained local interventionists (100%). The meta-analysis showed a 30% reduction in diarrhoea risk in the intervention group (RR = 0.69; 95% CI = 0.52–0.91, *P* = 0.01).

**Conclusions:**

Lifestraw Family water filters can be effective interventions to reduce diarrhoea in vulnerable paediatric populations for at least one year, though certain aspects related to physical environment and implementation may increase their public health impact. The findings of this study suggest considerations for scale-up that can be applied in settings in need of longer-term interim solutions until universal access to safe drinking water is achieved.

Diarrhoeal diseases are a leading cause of mortality in children under five years, claiming the lives of approximately 525 000 children each year [1]. Almost 90% of these deaths occur in sub-Saharan Africa and South Asia [2]. The pathogens responsible for diarrhoeal disease are often transmitted through consumption of faecally-contaminated water [3]. According to the World Health Organization (WHO)/United Nations Children’s Fund (UNICEF) Joint Monitoring Programme for Water Supply, Sanitation and Hygiene, over two billion people globally are at risk of diarrhoeal disease as a result of consuming faecally-contaminated water, and this risk is particularly high in low- and middle-income countries (LMICs), especially those in sub-Saharan Africa, which is home to half of the global population without access to an improved water source within 30 minutes roundtrip [4]. An improved water source refers to a water delivery point that is designed to protect water from faecal contamination (i.e. piped water, boreholes, protected wells and springs) [5].

United Nations Sustainable Development Goal (SDG) 6.1 aims to achieve universal safe drinking water access by 2030 [6], though progress is far off track in many LMICs, particularly in sub-Saharan Africa [4]. The ideal solution to universal safe water access involves piping treated water to all households, though the high cost of installation and maintenance means that this option may be decades away for many LMICs [7]. In the meantime, many living in these countries continue to collect water from improved or unimproved water sources and store it in the home, with contamination liable to occur at any stage along the pathway to consumption [8].

A recent study using Demographic and Health Survey data from 2013–2016 from 23 countries in sub-Saharan Africa found that only 18% of households used an appropriate water treatment method, with less than 1% using filters [9]. Acknowledging the increasing importance of household water treatment technologies in achieving SDG 6.1, the WHO established a standardised scheme to evaluate the performance of these technologies against common diarrhoea-causing pathogens, which was intended to help guide their selection by Member States and non-governmental organisations (NGOs) [10]. Lifestraw Family point-of-use household water filters were the first filters tested in the first round of this evaluation scheme that were found to provide comprehensive protection against many diarrhoea-causing pathogens [10]. The comprehensive protection rating means that these filters can be used in situations in which water quality is unknown or cannot be easily tested [10]. Lifestraw Family filters remove pathogens through gravitational force as water passes through hollow fibre membranes [10]. The first version, the Lifestraw Family 1.0, is a hanging filter with no built-in water storage, while the second version (Lifestraw Family 2.0) is a tabletop filter with 5.5 L of built-in safe water storage [10]. Both are low-cost options that treat a limited quantity of water, such as would be used in a household, and neither version requires electricity, making them appropriate for use in LMICs [10].

However, while these filters are considered effective water treatment options and are among the most common brands tested in intervention studies spanning over a decade, systematic reviews and meta-analyses to date have included very few studies, have not focused on the longer-term (≥12-month) effectiveness of these filters, and have not used a model that could inform scale-up strategies. A better idea of how and for how long these filters remain effective against diarrhoea could help inform scale-up strategies in areas that need longer-term interim solutions until universal safe water access is achieved.

## The model

**Figure 1:**
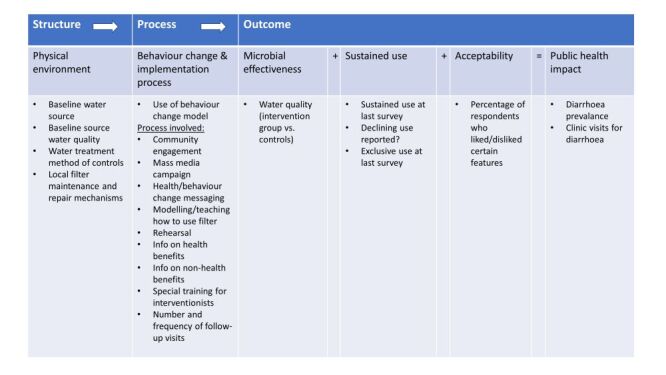
Donabedian Model of Care conceptualised to public health impact.

According to the WHO, the public health impact of water filters is dependent on: 1) the need for water filters based on water characteristics; 2) the effectiveness of the water filter in the context used; and 3) the correct and sustained use of water filters [11]. However, while these criteria reflect a linked step-wise process towards public health impact, they have never been integrated into a comprehensive model through which to guide a systematic review. One model that accounts for both the physical environment and the behaviour change and implementation process needed for sustained use is the Donabedian Model of Care [12]. This model, which considers the links between structure, process, and outcome, can be adapted to conceptualise how physical environment and behaviour change and implementation process might influence microbial effectiveness and sustained use, thus determining clinical effectiveness (**Figure 1**). Applying this model to individual studies through a systematic review could help build an evidence base to inform global health strategies.

## Physical environment

Water filters can have a public health impact when water treatment is needed and the filter is effective in the context used [11]. The WHO evaluated both versions of the Lifestraw Family filter under standardised conditions and found that both versions provided ‘comprehensive’ coverage. Of note, the Lifestraw Family 1.0 had slightly better microbial performance as it provided targeted protection against bacterial, protozoal, and viral pathogens, whereas the 2.0 filter offered targeted protection only against bacterial and viral pathogens, though health gains from the two versions are expected to be similar [10]. However, it is not known whether filter effectiveness against diarrhoea may differ according to the percentage of the study population using improved vs unimproved water sources or the baseline contamination of water when tested in field conditions. The percentage of controls who already treat water and the availability of local filter repair and maintenance are other factors related to the environment that could influence public health impact.

## Behaviour change and implementation process

In order to have public health impact, filter use must be sustained [13], though water treatment interventions have historically faced challenges in determining sustained behaviour change [14]. It is generally believed that behavioural change interventions are most effective when informed by theory [13]. However, a 2012 systematic review found that only 27% of studies used behaviour change theory to design and evaluate water treatment interventions [14]. Although progress has since been made in developing behaviour change models for water, sanitation, and hygiene (WASH) interventions [13], it can be difficult to understand what might work best for household filter interventions, which comprise only a small area under the WASH umbrella. According to the WHO, more behaviour change research is needed to optimise household water treatment uptake [15], though existing research suggests that complex interventions with high levels of community engagement are more successful than solely educational approaches [16].

## Public health impact

Public health impact can be conceptualised as microbial effectiveness and high sustained use, which lead to public health impact. Regarding microbial effectiveness, a prior systematic review found that no studies continually measured filters against the range of viral, bacterial, and protozoal pathogens that cause diarrhoea [17]. Regarding filter use, this same systematic review reported declining and non-exclusive use over time for water treatment interventions, which could potentially explain why this study found no evidence that household water treatment interventions reduced diarrhoea beyond 12 months [17].

However, regardless of these gaps, meta-analyses of filter interventions have generally reported promising results regarding public health impact, though none have exclusively focused on interventions with ≥12 months of follow-up. The most recent meta-analysis on household water filters reported a 50% reduction in diarrhoea risk for filters in general, but did not evaluate Lifestraw Family filters separately from other brands [18].

In light of these considerations, the aims of this systematic review were to: 1) report information on aspects related to physical environment and implementation that could impact clinical effectiveness, and 2) conduct an updated meta-analysis on Lifestraw Family filter effectiveness against diarrhoea based on studies that included ≥12 months of follow-up in children under five in LMICs. The Lifestraw Family filter was chosen because it was the first filter evaluated by the WHO in its water treatment evaluation scheme that provided comprehensive protection against many of the waterborne pathogens that cause diarrhoea and it has been one of the most common filter brands used in intervention studies spanning over a decade. In a recent meta-analysis, 30% of filter interventions after 2010 used the Lifestraw Family filter [18].

## METHODS

We conducted this systematic review according to Preferred Reporting Items for Systematic Reviews and Meta-Analyses (PRISMA) guidelines [19] (**Online Supplementary Document**).

### Search strategy

We conducted a literature search in November 2022 using a broad search strategy adapted for MEDLINE, Embase, Cochrane, and CINAHL databases (**Table 1**). The full inclusion criteria were: 1) RCTs, cluster-RCTs, quasi-experimental, or matched cohort studies on 2) Lifestraw Family 1.0 or 2.0 filters 3) conducted in LMICs 4) that evaluated filter effectiveness against diarrhoea in children under five and 5) analysed ≥12 months of follow-up data on clinical effectiveness against diarrhoea and were 6) published from 2010 with 7) full-text availability in English. We excluded studies if they failed to meet any of these criteria. Two researchers conducted the search and selected the included studies, while a third researcher was available in case of disagreement.

**Table 1:** Search terms and databases

	PubMed and Embase (via OVID)	Cochrane Library + Cochrane CENTRAL	CINAHL (via EBSCO)
1	exp water treatment/	water purification (MeSH)	MW water treatment
2	water treatment	water treatment	OR TX water treatment
3	membrane adj3 (filt* or purif*)	membrane NEAR/3 (filt* or purif*)	AND TX (membrane N3 (filt* or purif*))
4	Lifestraw	Lifestraw	OR TX Lifestraw
5	household adj3 (filt*or purif*)	household NEAR/3 (filt* or purif*)	OR TX (household N3 (filt* or purif*))
6	point-of-use adj3 (filt* or purif*)	point-of-use NEAR/3 (filt* or purif*)	OR TX (point-of-use adj3 (filt* or purif*))
7	hollow fib* adj3 (ultrafit*)	hollow fib* NEAR/3 ultrafilt*	OR TX hollow fib* N3 ultrafilt*
8	hollow fib* adj3 (filt* or purif*)	hollow fib* NEAR/3 (filt* or purif*)	OR TX (hollow fib* N3 (filt* or purif*))
9	exp diarrhea/	Diarrhea (MeSH)	AND MW diarrh#ea
10	diarrh?ea	diarrhoea*	AND TX diarrh#ea
11	#1 or #2	diarrhea*	publication date from Jan 2010 to Nov 2022
12	#3 or #4 or #5 or #6 or #7 or #8	#1 or #2	
13	#9 or #10	#3 or #4 or #5 or #6 or #7 or #8	
14	#11 and #12	#9 or #10 or #11	
15	#12 and #13	#12 and #13	
16	#14 or #15	#13 and #14	
17	#15 or #16 with publication date from Jan 2010 to Nov 2022	#15 or #16 with publication date from Jan 2010 to Nov 2022	

### Data extraction

#### Trial data

One researcher extracted trial characteristics, which were confirmed by a second researcher. Extracted data included study design, intervention and control group definitions, primary and secondary outcome measures, diarrhoea outcome measure, recall period, and case definition, and study duration (**Table 2**).

**Table 2:** Trial characteristics of included studies

Study	Haque et al., 2022	Fagerli et al., 2020	Kirby et al., 2019	Kirby et al., 2017	Peletz et al., 2012	Boisson et al., 2010
Study design	Cluster randomised	Cluster randomised	Cluster randomised	Matched cohort	Individual randomised	Individual randomised
Setting	Rwanda	Kenya	Rwanda	Rwanda	Zambia	Democratic Republic of Congo
Age group targeted and number of children	Under five years; 1483 (759 intervention; 724 control)	Under 12 mo; 166 (92 intervention; 74 control)	Under five years; 2440 (1190 intervention; 1250 control)	Under five years; 340 (147 intervention, 193 control)	Under two years; 121 (61 intervention; 60 control)	Under five years; 190 (85 intervention; 105 control)
Interventiongroup intervention	Lifestraw Family filter 2.0	Lifestraw Family filter 1.0	Lifestraw Family filter 2.0	Lifestraw Family filter 2.0	Lifestraw Family filter 1.0 + 2.5-L water storage containers	Lifestraw Family filter 1.0
Control group intervention	Community-Based Environmental Health Promotion Programme (CBEHPP) alone	Nyando Integrated Child Health and Integration Project alone	No intervention	No intervention	No intervention	Controls received placebo filter that did not remove protozoa, viruses, or bacteria
Additional active components of intervention or background intervention	CBEHPP	Nyando Integrated Child Health and Integration Project	Cookstoves provided, ‘Tubeho Neza’ community and household education and materials	Cookstoves provided, ‘Tubeho Neza’ community and household education and materials	None	None
Primary outcome	*Escherichia coli* in drinking water	Diarrhoea prevalence	Diarrhoea prevalence	Household drinking water quality (thermotolerant coliforms)	Filter use, drinking water quality, diarrhoea prevalence, weight-for-age z scores	Diarrhoea prevalence
Secondary outcome	Filter adoption, diarrhoea prevalance, health care visits for diarrhoea		Healthcare visits for diarrhoea, prevalence of bloody dysentery and persistent diarrhoea, acute respiratory infection prevalence and health care visits	Diarrhoea prevalence, filter coverage and use, health care visits for diarrhoea		Water quality, filter use
Diarrhoea outcome measure	Caregiver-reported diarrhoea, health care visits	Caregiver-reported diarrhoea	Caregiver-reported diarrhoea, health care visits	Caregiver-reported diarrhoea, health care visits	Caregiver-reported diarrhoea	Caregiver-reported diarrhoea
Recall period	7 d	48 h	7 d	7 d	7 d	7 d
Diarrhoea case definition	WHO standard definition of 3 or more loose stools in 24 h	WHO standard definition of 3 or more loose stools in 24 h	WHO standard definition of 3 or more loose stools in 24 h	WHO standard definition of 3 or more loose stools in 24 h	WHO standard definition of 3 or more loose stools in 24 h	WHO standard definition of 3 or more loose stools in 24 h
Study duration	16 mo	12 mo	12 mo	24 mo	12 mo	12 mo

WHO – World Health Organization

#### Donabedian Model of Care data

Consistent with the conceptualisation of public health impact according to the Donabedian Model of Care as a linked stepwise process (**Figure 1**), one researcher extracted data from each study on: 1) physical environment (water source, baseline water quality, water treatment method used by controls, local filter maintenance), 2) behaviour change and implementation process (use of behaviour change theory, implementation steps, special training of interventionists), and 3) outcome (clinical effectiveness, water quality, sustained use, exclusive use, and acceptability at last follow-up). A second researcher confirmed the extracted data.

### Meta-analysis

For the meta-analysis, we collected data on diarrhoea prevalence by extracting adjusted longitudinal prevalence ratios, prevalence ratios, risk ratios, or odds ratios (ORs) and confidence intervals (CIs) from all studies. If a study reported diarrhoea prevalence in various subgroups under age five (e.g. <2 and <5 years), the ratio extracted was the one from the age group targeted for recruitment. Since ORs may distort risk when the outcome is not rare, ORs and their CIs were converted to relative risk (RR) when incidence in the control group was reported using the method described by Zhang and Yu [20]. If studies reported credible intervals, CIs were obtained from *P*-values using the method described by Altman and Bland [21]. Standard errors for all effect estimates were calculated according to the method described by Higgins et al. [22].

### Statistical analysis

We used descriptive statistics (frequency counts) to analyse trial and Donabedian Model of Care data.

We conducted a random-effects meta-analysis to obtain the pooled effect size of the Lifestraw Family filter in reducing diarrhoea in children under five using SPSS version 28 (IBM Corp., Armonk, NY, USA). The χ^2^ test assessed heterogeneity between study estimates, while the *I^2^* test evaluated the percentage of variability in effect estimates due to heterogeneity. A funnel plot assessed publication bias.

### Risk of bias

We used a modified Newcastle-Ottawa scale [23] as used in a previous systematic review [18] to assess risk of bias. This scale includes seven domains, including selection bias, response bias, follow-up bias, misclassification bias, outcome assessment bias, outcome measurement bias, and bias in analysis, and has a maximum score of 9. Two researchers confirmed the scores assigned, with a third available in case of disagreement.

## RESULTS

**Figure 2:**
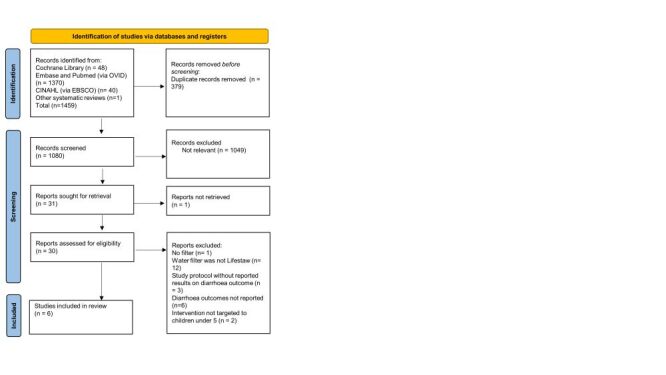
Preferred Reporting Items for Systematic Reviews and Meta-analyses (PRISMA) flowchart of included studies.

**Figure 2** shows the PRISMA flowchart of included studies. Five studies plus an additional study that had a mean follow-up of 11.2 months met the inclusion criteria and were included. These six studies included a total of 4740 children under five years. Two studies used the Lifestraw Family 1.0 filter, one study used the Lifestraw Family 1.0 filter with additional water storage containers, and three studies used the Lifestraw 2.0 filter [24–29]. Trial characteristics are presented in **Table 2**.

### Systematic review

Regarding physical environment (**Table 3**), studies that reported filter effectiveness against diarrhoea generally had a sizable proportion of the population that used improved water sources and a majority proportion of controls who did not treat water. Of the four studies that reported significant results, three reported that more than 70% of the study population had access to improved water and all four studies reported that less than 50% of controls currently treated water. Three out of four interventions that reported significant reductions in diarrhoea risk involved community health workers in the maintenance and repair of broken filters.

**Table 3:** Physical environment, behaviour change and implementation process, and outcome of studies

Physical environment						
Study	Haque et al., 2022	Fagerli et al., 2020	Kirby et al., 2019	Kirby et al., 2017	Peletz et al., 2012	Boisson et al., 2010
Intervention	Lifestraw Family filter 2.0	Lifestraw Family filter 1.0	Lifestraw Family filter 2.0	Lifestraw Family filter 2.0	Lifestraw Family filter 1.0 + 2 5-L water storage containers	Lifestraw family filter 1.0
Baseline water source	Improved – 89.1% intervention group/86.6% control	Improved – 49% intervention group/41.1% control	Improved – 77.9% intervention group/74.3% control	Improved – 92.9% intervention group/85.9% control	Improved – 41% intervention group/53% control	Majority relied on unimproved water
Baseline source water characteristics	Mean *Escherichia coli* = 207.4 CFU in intervention group/215.4 in control	Not reported	34.9% of intervention group had thermotolerant coliform (TTC) levels >100/100 mL; 37.7% controls	99% of intervention group had TTC levels <101/100 mL, 89.2% controls	Source TTC geometric mean – 117 (95% confidence interval (CI) = 72–190) intervention/193 (95% CI = 114–328) control	75% of household samples showed >1000 TTC/100 ml
Water treatment method used by controls	47.3% treated water	91.8% treated water, with 95.5% using chlorination	16.8% treated water	42.6% of matched villages treated water	22% treated water	2.5% treated water
Local filter maintenance and repair	Local Community Health/Hygiene Club facilitators were main service providers and were trained to repair and replace filters	Unknown, fieldworkers replaced broken filters within two days	Community health workers trained to repair filters and resolve maintenance issues	Community health workers trained to repair filters and resolve maintenance issues	Unknown, broken filters were replaced	Unknown, broken filters were replaced
**Behaviour change and implementation process**						
Reported use of behaviour change model?	Yes – Diffusion of Innovation theory; Health Beliefs Model	Not reported	Yes – Diffusion of Innovation theory; Health Beliefs Model	Yes – Diffusion of Innovation theory; Health Beliefs Model	Not reported	Not reported
Process involved						
Community engagement	Yes – Community Health Club (CHC) weekly meetings that reinforced filter use; integration into existing local programme	Yes – community meeting to distribute filter and teach filter use	Yes – community meetings to reinforce behaviour change; integration into existing local programmes	Yes – community meetings to reinforce behaviour change; integration into existing local programmes	Not reported	Not reported
Mass media campaign	Not reported	Not reported	Yes	Yes	Not reported	Not reported
Health/behaviour change messaging	Yes	Not reported	Yes	Yes	Not reported	Not reported
Modelling/teaching how to use filter	Yes	Yes	Yes	Yes	Yes	Not reported
Rehearsal	Yes	Not reported	Yes	Yes	Not reported	Not reported
Info on health benefits	Yes	Not reported	Yes	Yes	Not reported	Not reported
Info on non-health benefits	Not reported	Not reported	Yes	Yes	Not reported	Not reported
Health adopters	Not reported	Not reported	Yes	Yes	Not reported	Not reported
Did interventionists receive special training on intervention?	Yes – CHC facilitators received 3-d training on filter use, maintenance, household education, and behaviour change	Not reported	Yes – community health workers (CHWs) received 2.5-d training on filter use, maintenance, surveys, data collection, household engagement	Yes – CHWs received 2.5-d training on filter use, maintenance, surveys, data collection, household engagement	Yes, from filter manufacturer	Not reported
Number and frequency of follow-up visits	2 visits by CHC facilitators – 1 to teach and 1 to reinforce filter use; 2 health survey follow-ups at 5–7 and 13-16 mo	Biweekly follow-up visits for 12 mo	3 follow-up visits conducted at 4-mo intervals	Two follow-up visits at 12-18 mo and at 19-24 mo	Monthly follow-up visits for up to 12 mo	Monthly follow-up visits for 12 mo
**Outcome**						
Diarrhoea prevalence	Prevalence ratio (PR) = 0.51 (95% CI = 0.35–0.73)	Odds ratio (OR) = 1.19 (95% CI = 0.74–1.90)	PR = 0.71 (95% CI = 0.59–0.87)	OR = 0.50 (95% credible interval = 0.23–0.90)	Longitudinal prevalence ratio (LPR) = 0.47 (95% CI = 0.30–0.73)	LPR = 0.85 (95% CI = 0.56–1.28)
Diarrhoea clinic visits	PR = 0.46 (95% CI = 0.22–0.96) (7-d)	Not reported	PR = 0.54 (95% CI = 0.32–0.91) (7-d)	OR = 0.60 (95% credible interval = 0.27–1.11) (3 mo)	Not reported	Not reported
Water quality	Any *Escherichia coli* contamination (≥2 CFU/100 mL): PR = 0.80 (95% CI = 0.74–0.87)	*Escherichia coli* contamination in stored water: OR = 0.42 (95% CI = 0.24–0.74)	Detectable thermotolerant coliform (TTC) in drinking water: PR = 0.62 (95% CI = 0.57–0.68)	Detectable TTC in drinking water: OR = 0.22 (95% credible interval 0.10–0.39)	3 vs 181 TTC/100 mL in intervention and control households respectively, *P* < 0.001	Active filter log TTC reduction of 2.98 (95% CI = 2.88–3.08), placebo filter 1.05 (95% CI = 0.93–1.16)
Sustained use at last survey	Participants: -reported filter currently used (93.6%); -reported filter filled in last 7 d (91.6%); -had a filter storage with water in it (75%); -reported that drinking water taken was treated with filter (80.7%); -reported that one young child drank filtered water yesterday (78.8%)	-Visibly wet filter at visit (45.5%); -less than 25% of intervention households reported treating their water on the day of the follow-up visit	Participants: -had filter observed in household and reported it was filled yesterday (47.8%); -had filter observed in household and reported it was filled the day before yesterday (71.1%); -had a filter that looked to be in use (64.8%); -had a filter containing water (54.4%)	Participants: -reported currently using filter (98.7%); -reported filter was used on day of visit or previous day (88.2%); -had filter that looked to be in use (90.8%); -had filter containing water (81.6%); -had a filter that was filled on at least half of the days for which sensor data was available, (36.8%)	92% of participants were confirmed users at last follow-up visit	75.8% of participants in intervention group reported using filter the previous day
Declining use from last survey?	Yes	Not reported	Yes	Yes	No	No
Exclusive use at last survey	Not reported	Not reported	Not reported	Participants: -reported children <5 drank only filtered water today or yesterday (91.8%); -reported children <5 always drank filtered water when home (66.6%); -reported children <5 always drank filtered water when not home (66.6%)	96% of children under 2 drank only filtered water that day or the day prior	4.9% of children under 5 drank only filtered water the previous day (reported at 8-mo follow-up)
Acceptability	Percentage of sample who reported the following features as being very acceptable or acceptable: -appearance (100%) -taste (99.6%) -smell (99.5%) -time to filter water (88.2)	Participants dislike slow flow, small size of the reservoir, and need to hang the filter. Reasons for non-use: -flow too slow (74%) -takes too long (63%) -inflow reservoir too small (61%) -other methods treat more water (58%) or are safer (35%) -children cannot use alone (33%)	N/A	N/A	Participants liked filter because it: -provided safe water, 75%; -improved taste, 13%; -provided good water, 9%; -was easy to use, 2%; participants dissatisfied with: -nothing, 100%	Participants liked filter due to improved: -aesthetics, 88%; - taste, 92%; -odour, 56%; -health, 35%; participants dissatisfied with: -low flow rate, 87%; -small top container, 85%; -problems with rats, 44%.

Regarding behaviour change and implementation process (**Table 3**), three studies reported using behaviour change theory to guide implementation, and all three reported significant results. As for implementation steps, nearly all studies reported modelling the water filter or teaching participants how to use it. However, three out of four studies that reported significant results also included other implementation components, including community engagement, health/behaviour change messaging campaigns, participant rehearsal of how to use and maintain the filter, and information on health benefits. All four interventions that reported significant results also reported that interventionists received special training on filter use and maintenance, with three reporting that this training targeted community health workers and encompassed participant engagement and education.

In terms of outcome (**Table 3**), all interventions evaluated filter effectiveness against one bacterial pathogen. Four studies measured filter performance in terms of thermotolerant coliform (TTC) reduction, while two evaluated effectiveness against *Escherichia coli*. All studies found significant differences with respect to the control group. Regarding sustained use, studies differed widely in how this was assessed. Investigating exclusive use in children under five identified important nuances in overall use. For example, one study that reported sustained household use of nearly 76% also reported that <5% of children drank only filtered water the previous day. Exclusive use in children was assessed in only three studies and ranged from less than 5% to as much as 96%. Of these three studies, exclusive use was reported to be higher than 90% on some measures in the two studies that reported significant clinical results. Of the four studies that reported acceptability, acceptability scores were generally predictive of sustained use and clinical effectiveness.

The two studies with predominately positive acceptability ratings used the Lifestraw Family 1.0 filter with additional water storage and the Lifestraw Family 2.0 filter and reported high sustained use and significant diarrhoea reduction. In contrast, the other two studies used the Lifestraw Family 1.0 without additional water storage and reported a large percentage of participants who were unhappy with certain features, with these studies also reporting low sustained use and non-significant diarrhoea results.

### Overall conceptualisation of the Donabedian Model of Care

In summary, there seemed to be relevant links between physical environment (use of the Lifestraw Family 2.0 filter or the 1.0 filter with additional safe water storage, improved water access, and local filter maintenance and repair mechanisms), behaviour change and implementation process (use of behavaiour change theory, a multifaceted implementation process, and specially-trained interventionists), and the outcome measures of sustained use and acceptability, which in turn contributed to clinical effectiveness.

### Meta-analysis

**Figure 3:**
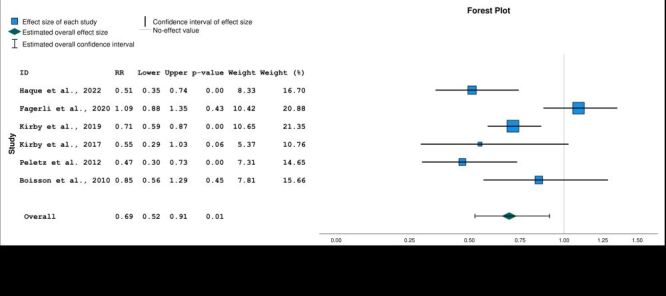
Forest plot of relative risk of caregiver-reported diarrhoea with Lifestraw Family 1.0 and 2.0 filters. Model: Random-effects model. Heterogeneity: Tau-squared = 0.08, H-squared = 4.20, *I*-squared = 0.76; Homogeneity: Q = 21.27, df = 5, *P* = 0.01. Test of overall effect size: z = −2.60, *P* = 0.01. Axis is shown using log scale.

**Figure 4:**
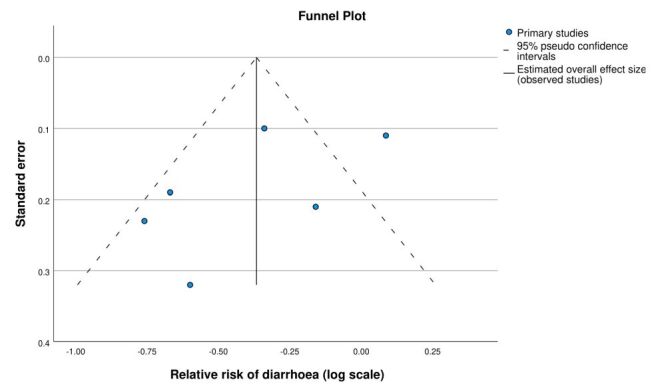
Funnel plot of studies reporting caregiver-reported diarrhoea with Lifestraw Family 1.0 and 2.0 filters.

Six studies that evaluated caregiver-reported diarrhoea in children under five were included in a random-effects meta-analysis. The meta-analysis showed an overall 31% reduction in risk of diarrhoea in the Lifestraw Family intervention group (pooled RR = 0.69; 95% CI = 0.52–0.91, *P* = 0.01) (**Figure 3**). Heterogeneity of included studies was high (*I^2^* = 0.76). A funnel plot did not suggest any publication bias (**Figure 4**).

**Figure 5:**
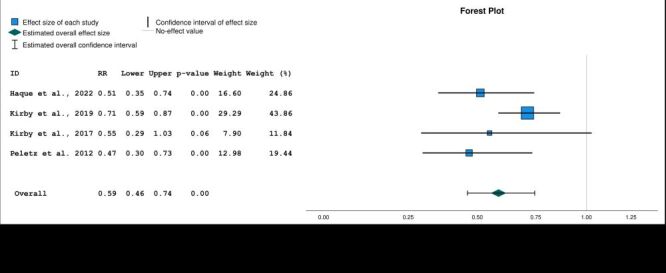
Sensitivity analysis, diarrhoea relative risk when removing Lifestraw 1.0 interventions with no additional water storage. Model: Random-effects model. Heterogeneity: Tau-squared = 0.02, H-squared = 1.68, *I*-squared = 0.41; Homogeneity: Q = 4.55, df = 3, *P* = 0.21. Test of overall effect size: z = −4.37, *P* = 0.00. Axis is shown using log scale.

Since the systematic review indicated that interventions that used the Lifestraw Family 1.0 filter with no additional water storage containers were less clinically effective than those that included water storage, we conducted a sensitivity analysis that excluded Lifestraw Family 1.0 interventions that did not include additional water storage. This sensitivity analysis resulted in a larger reduction in diarrhoea risk (RR = 0.41; 95% CI = 0.46–0.74, *P* < 0.01) and an improved *I^2^* value of 0.41 (**Figure 5**).

**Figure 6:**
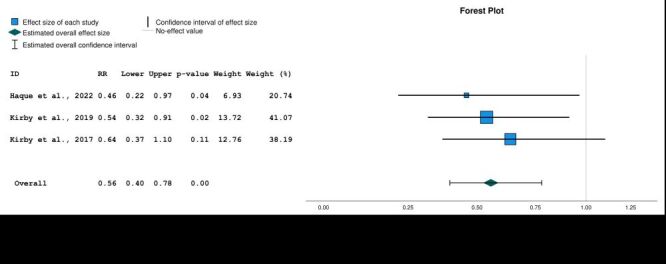
Relative risk of clinic visits for diarrhoea. Model: Random-effects model. Heterogeneity: Tau-squared = 0.00, H-squared = 1.00, *I*-squared = 0.00; Homogeneity: Q = 0.51, df = 2, *P* = 0.77. Test of overall effect size: z = −3.40, *P* = 0.00. Axis is shown using log scale.

Three studies also reported diarrhoea as measured by caregiver-reported clinic visits for diarrhoea. The pooled RR for the intervention group was 0.56 (95% CI = 0.40–0.78, *P* < 0.01) and the *I^2^* value was 0 (**Figure 6**).

### Risk of bias

All studies involved limitations related to missing follow-up data and the use of self-report outcome measures, and five out of six had non-blinded study designs (**Online Supplementary Document**). Four non-blinded studies received a score of 4/9 and one received a score of 5/9. Of non-blinded studies, all had follow-up, outcome assessment, and outcome measurement bias, and two also had response bias. Two studies accounted for response bias with the use of a negative control (toothache). The blinded study received a score of 6/9, reflecting follow-up and outcome assessment bias.

## DISCUSSION

This study indicates that Lifestraw Family filters are an effective longer-term interim solution to combat diarrhoea in children under five in sub-Saharan Africa. The finding that these filters may reduce diarrhoea by at least 30% is consistent with previous evidence on Lifestraw filters [17]. However, the meta-analysis by Clasen et al. only included three studies, only two of which used the Lifestraw Family filter [17]. These results contrast with those of a meta-analysis published in 2017 that found no significant effect of Lifestraw filters [30], though that subgroup analysis only included two studies, only one of which used the Lifestraw Family filter.

With respect to existing literature, the main strengths of this study are 3-fold. First, this study provided an updated meta-analysis on a household filter with comprehensive protection against many diarrhoea-causing pathogens as evaluated by WHO standards [11]. Second, this study extracted information on physical environment, behaviour change and implementation process, microbial effectiveness, filter use, and acceptability, allowing clinical effectiveness results to be interpreted in light of these aspects. Third, it included only studies with ≥12 months of follow-up, thus providing important information on the feasibility of these filters in settings that require longer-term interim solutions until universal access to piped drinking water is achieved.

The most significant limitation of this study was the unblinded study design of five out of six studies, which resulted in a high risk of bias. Unblinded studies result in exaggerated effect estimates [31] and may influence the subjective reporting of outcome measures [32]. Unfortunately, even the blinded study found that blinding was unfeasible since the placebo filter removed pathogens in field conditions [29]. In addition, all studies were subject to recall and courtesy bias, and the effect of observation may have increased water treatment behaviour (Hawthorne effect). Heterogeneity in the main analysis was high (*I^2^* = 0.76), though the sensitivity analysis resulted in a reduced *I^2^* of 0.41. However, a funnel plot for the main analysis did not suggest any publication bias, though the small number of studies did not allow a definitive conclusion. The supplementary analysis of caregiver-reported clinic visits for diarrhoea found that the filter significantly reduced potentially more severe cases of diarrhoea, which may be less subject to recall bias, and had no heterogeneity concerns, but the small number of studies included (n = 3) was a limitation.

Application of the Donabedian Model of Care highlighted several aspects for consideration. Regarding physical environment, results suggest that health impact is greater in environments where a large proportion of the population uses improved water sources and no water treatment method. The finding regarding improved water access supports an existing hypothesis that less turbidity in improved water sources results in a faster flow rate and thus greater acceptability and sustained use [24]. Results also suggest it may be beneficial to strengthen local filter maintenance and repair mechanisms by training community health workers, a finding consistent with the Integrated Behavioural Model for Water, Sanitation, and Hygiene, which advocates WASH technology maintenance at the community level [14].

Regarding behaviour change and implementation process, this study contributes to existing literature through its findings that interventions with clinical benefit were frequently based on behaviour change theory and included community engagement, health messaging campaigns, modelling/teaching and rehearsal of filter use and maintenance, information on health benefits, and a prominent role of community health workers, who received special training on household engagement and filter use, maintenance, and repair. These findings are consistent with WHO recommendations for WASH behaviour change interventions, which encourage multiple intervention components informed by behaviour change theory and high levels of community leadership and engagement [16]. Studies that used a solely educational approach did not report significant health results, with the exception of the study by Peletz et al. [28]. However, this study included children of HIV-positive mothers who were recruited from health clinics and who may have been extra motivated to use the filter.

Regarding microbial outcome, all studies reported significant improvements in water quality as measured by TTC or *Escherichia coli*. However, these methods only measure bacterial contamination, not viral or protozoal contamination [33].

Regarding filter use, of the three studies that reported exclusive use, the two with clinically significant results reported exclusive use over 90% at last survey, a figure consistent with the minimum threshold proposed by the WHO to obtain health impact [11]. Indeed, even occasionally drinking unfiltered water can significantly increase diarrhoea risk [34]. Only four studies reported on acceptability, but those that did evidenced greater satisfaction with the Lifestraw Family 2.0 filter or the 1.0 filter with additional safe water storage, and this increased acceptability was associated with higher sustained use and clinical effectiveness. This result, together with the sensitivity analysis, suggests that scale-up should prioritise use of the Lifestraw Family 2.0 filter or 1.0 filter with additional water storage.

According to the WHO, water treatment methods offering comprehensive protection against diarrhoea-causing pathogens, such as Lifestraw Family filters, are appropriate in all settings, including those in which water microbial quality is unknown [11]. However, gaps remain regarding the best way to implement these interventions at large scale. Studies using the Lifestraw Family 2.0 filter and an implementation process characterised by use of behaviour change theory, community engagement, health messaging, local filter repair-and-replace mechanisms, and specially-trained local interventionists have placed the cost of these filters at between 30–40 US dollars and the cost of distribution and programmatic support for a two-year period at between 14–80 US dollars, with the majority of costs sustained in the first year [35,36].

One innovative way to finance this type of intervention has been within the context of carbon offset programmes, where Lifestraw filters have been promoted as a zero-carbon-emission water treatment technology eligible for carbon credit financing [37]. Carbon credits are claimed by calculating the amount of greenhouse gas emissions avoided through the use of zero-carbon-emission water treatment technology as opposed to the alternative water treatment option of boiling water with fossil fuels or non-renewable energy [38]. Carbon credit programmes have the potential to fund large-scale implementation and incentivise long-term use [38].

However, a previous study by Pickering et al. found very low sustained filter use over the long term (two to three years) in this context and questioned whether it might be better to implement zero-carbon-emission water treatment technology at the community rather than household level [39]. The present systematic review instead lends support to the feasibility of household-level water treatment technology implementation in determining sustained behaviour change and health benefits over at least 12 months and up to 24 months, and suggests considerations for scale-up that can be used in the context of carbon offset programmes. Indeed, the study by Pickering et al. assessed use of the Lifestraw Family 1.0 filter [39], when results of this study suggest that the 2.0 filter or the 1.0 with additional water storage may have better acceptability and sustained use.

## CONCLUSIONS

In conclusion, this study provides promising results regarding the ability of Lifestraw Family filters to combat childhood diarrhoea in LMICs for at least 12 months, though it was not able to address how long these filters may remain effective. Future large-scale studies are needed to confirm and expand these findings, ideally with the use of at least two to three years of follow-up, less subjective health outcome measures, a wider range of microbial testing to also encompass protozoal and viral contamination over follow-up, and more standardised reporting of sustained exclusive use.

## Additional material


Online Supplementary Document
