# Supplementary material for: Lifestraw Family water filters in low- and middle-income countries: a systematic review and meta-analysis to define longer-term public health impact against childhood diarrhoea and inform scale-up
Source: J Glob Health. 2024 Jun 28;14:04018. doi: 10.7189/jogh.14.04018 (PMC11215800; doi:10.7189/jogh.14.04018)
Supplement: Online Supplementary Document [file jogh-14-04018-s001.pdf]

Section and Topic	Item #	Checklist item	Location where item is reported
**TITLE**			
Title	1	Identify the report as a systematic review.	Title
**ABSTRACT**			
Abstract	2	See the PRISMA 2020 for Abstracts checklist.	Abstract
**INTRODUCTION**			
Rationale	3	Describe the rationale for the review in the context of existing knowledge.	'Physical environment', 'behaviour change and implementation process', and 'public health impact' paragraphs
Objectives	4	Provide an explicit statement of the objective(s) or question(s) the review addresses.	Last paragraph of introduction
**METHODS**			

Section and Topic	Item #	Checklist item	Location where item is reported
Eligibility criteria	5	Specify the inclusion and exclusion criteria for the review and how studies were grouped for the syntheses.	‘Search strategy’ paragraph
Information sources	6	Specify all databases, registers, websites, organisations, reference lists and other sources searched or consulted to identify studies. Specify the date when each source was last searched or consulted.	‘Search strategy’ paragraph
Search strategy	7	Present the full search strategies for all databases, registers and websites, including any filters and limits used.	Table 1
Selection process	8	Specify the methods used to decide whether a study met the inclusion criteria of the review, including how many reviewers screened each record and each report retrieved, whether they worked independently, and if applicable, details of automation tools used in the process.	‘Search strategy’ paragraph
Data collection process	9	Specify the methods used to collect data from reports, including how many reviewers collected data from each report, whether they worked independently, any processes for obtaining or confirming data from study investigators, and if applicable, details of automation tools used in the process.	‘Data extraction’ paragraphs
Data items	10a	List and define all outcomes for which data were sought. Specify whether all results that were compatible with each outcome domain in each study were sought (e.g. for all measures, time points, analyses), and if not, the methods used to decide which results to collect.	‘Data extraction’ paragraphs
	10b	List and define all other variables for which data were sought (e.g. participant and intervention characteristics, funding sources). Describe any assumptions made about any missing or unclear information.	‘Data extraction’ paragraphs

Section and Topic	Item #	Checklist item	Location where item is reported
Study risk of bias assessment	11	Specify the methods used to assess risk of bias in the included studies, including details of the tool(s) used, how many reviewers assessed each study and whether they worked independently, and if applicable, details of automation tools used in the process.	'Risk of bias' paragraph
Effect measures	12	Specify for each outcome the effect measure(s) (e.g. risk ratio, mean difference) used in the synthesis or presentation of results.	'Meta-analysis' paragraph
Synthesis methods	13a	Describe the processes used to decide which studies were eligible for each synthesis (e.g. tabulating the study intervention characteristics and comparing against the planned groups for each synthesis (item #5)).	Eligibility criteria in 'Search strategy' paragraph
	13b	Describe any methods required to prepare the data for presentation or synthesis, such as handling of missing summary statistics, or data conversions.	'Meta-analysis' paragraph
	13c	Describe any methods used to tabulate or visually display results of individual studies and syntheses.	'Statistical analysis' paragraph
	13d	Describe any methods used to synthesize results and provide a rationale for the choice(s). If meta-analysis was performed, describe the model(s), method(s) to identify the presence and extent of statistical heterogeneity, and software package(s) used.	'Statistical analysis' paragraph
	13e	Describe any methods used to explore possible causes of heterogeneity among study results (e.g. subgroup analysis, meta-regression).	N/A

Section and Topic	Item #	Checklist item	Location where item is reported
	13f	Describe any sensitivity analyses conducted to assess robustness of the synthesized results.	N/A
Reporting bias assessment	14	Describe any methods used to assess risk of bias due to missing results in a synthesis (arising from reporting biases).	Statistical analysis paragraph
Certainty assessment	15	Describe any methods used to assess certainty (or confidence) in the body of evidence for an outcome.	N/A
**RESULTS**			
Study selection	16a	Describe the results of the search and selection process, from the number of records identified in the search to the number of studies included in the review, ideally using a flow diagram.	PRISMA flowchart (Fig. 2)
	16b	Cite studies that might appear to meet the inclusion criteria, but which were excluded, and explain why they were excluded.	N/A
Study characteristics	17	Cite each included study and present its characteristics.	Table 2
Risk of bias in studies	18	Present assessments of risk of bias for each included study.	'Risk of bias' paragraph and online supplementary document

Section and Topic	Item #	Checklist item	Location where item is reported
Results of individual studies	19	For all outcomes, present, for each study: (a) summary statistics for each group (where appropriate) and (b) an effect estimate and its precision (e.g. confidence/credible interval), ideally using structured tables or plots.	Fig. 3- Forest plot
Results of syntheses	20a	For each synthesis, briefly summarise the characteristics and risk of bias among contributing studies.	'Risk of bias' paragraph
	20b	Present results of all statistical syntheses conducted. If meta-analysis was done, present for each the summary estimate and its precision (e.g. confidence/credible interval) and measures of statistical heterogeneity. If comparing groups, describe the direction of the effect.	Fig. 3
	20c	Present results of all investigations of possible causes of heterogeneity among study results.	Fig. 5- sensitivity analysis
	20d	Present results of all sensitivity analyses conducted to assess the robustness of the synthesized results.	Fig. 5- sensitivity analysis
Reporting biases	21	Present assessments of risk of bias due to missing results (arising from reporting biases) for each synthesis assessed.	Fig. 4
Certainty of evidence	22	Present assessments of certainty (or confidence) in the body of evidence for each outcome assessed.	Not performed- see para. 3 of Discussion for critical

Section and Topic	Item #	Checklist item	Location where item is reported
			interpretation of results
**DISCUSSION**			
Discussion	23a	Provide a general interpretation of the results in the context of other evidence.	Paragraphs 1-2, 4-7
	23b	Discuss any limitations of the evidence included in the review.	Paragraph 3
	23c	Discuss any limitations of the review processes used.	Paragraph 3
	23d	Discuss implications of the results for practice, policy, and future research.	Paragraphs 8-10
**OTHER INFORMATION**			
Registration and protocol	24a	Provide registration information for the review, including register name and registration number, or state that the review was not registered.	'Review protocol and registration' paragraph
	24b	Indicate where the review protocol can be accessed, or state that a protocol was not prepared.	'Review protocol and registration' paragraph

Section and Topic	Item #	Checklist item	Location where item is reported
	24c	Describe and explain any amendments to information provided at registration or in the protocol.	N/A
Support	25	Describe sources of financial or non-financial support for the review, and the role of the funders or sponsors in the review.	‘Funding’ section
Competing interests	26	Declare any competing interests of review authors.	‘Disclosure of interest’ section
Availability of data, code and other materials	27	Report which of the following are publicly available and where they can be found: template data collection forms; data extracted from included studies; data used for all analyses; analytic code; any other materials used in the review.	Methods and tables

**Reference**	**Risk of bias assessment for intervention studies**			-	-	-	-	-
	**Selection bias**	**Response bias**	**Follow-up bias**	**Misclassification bias**	**Outcome assessment bias**	**Outcome measurement bias**	**Bias in analysis**	**Total**
	Is there evidence of selection bias, which refers to systematic differences between baseline characteristics of the groups that are compared? Please specify as either yes, possible or no (= 1 star), with details. **(c)RCTs receive 1 star, unless evidence of selection bias (e.g. randomisation procedures not followed). Meaningful differences between groups at baseline in (c)RCTs receive 0 stars. Rates of declining to participate >10% receive 0 stars. Non- or quasi-randomised studies receive 0 stars.**	*Is there evidence of response bias? Please specify as either yes, possible or no (= 1 star), with details.* **If intervention recipient was not blinded to intervention status, 0 stars.**	*Is there evidence of bias due to missing follow-up data? Please specify as either yes, possible or no (= 1 star), with details (specifying the amount of loss to follow-up).* **<10% receives 1 star, ≥10% receives 0 stars.**	*Is there risk of households not receiving the intervention being misclassified as having received it, or vice versa? Please specify as either yes, possible, or no (=1 star), with details.* **Interventions delivered at the household/individual level receive 1 star. Interventions delivered at the community level that missed a substantial, i.e. ≥10%, proportion of the target population receive 0 stars, including when there is insufficient information to verify whether this is the case. Interventions with substantial risk of contamination (control households receiving intervention) receive 0 stars.**	*Is there evidence of bias arising from how the outcome was assessed? Please specify as either yes, possible, or no (=1 star), with details.* **Parent / person recall (=0 stars). Fieldworker assessed (=1 star). Physician / microbiologically assessed (=2 stars)**	*Is there evidence of ascertainment bias? Please specify as either yes, possible or no (= 1 star).* ***If outcome measurement staff were not blinded to intervention status, 0 stars.***	*Is there evidence that analysis was not appropriately adjusted for clustering and/or confounding, if appropriate? Please specify as either yes, possible or no.* **Scoring is based on losing stars (max. 2). Individual RCTs with baseline balance on covariates are unlikely to require adjustment (=2 stars). Cluster-RCTs and non-randomised trials may require adjustment for clustering (-1 star if not done). RCTs or cRCTs may require adjustment for covariates, with justification (-1 star if not done). Non-randomised studies require adjustment for covariates (-1 star if not done), but also adequate justification for covariate selection (-1 star if not included) – nb. there can be "too few" or "too many" covariates.**	

Haque et al., 2022	1	1, negative control used	0, 88% and 89% of possible diarrhoea week data in children under 5 were analysed in intervention and control groups, respectively.	1, at the household level	0, caregiver recall	0, assessors/participants not blinded	2	**5**
Fagerli et al., 2020	1	0, non-blinded	0, missing data from 22.7% of households in the intervention group and 29.5% of households in the comparison group	1, at the household level	0, caregiver recall	0, assessors/participants not blinded	2	**4**
Kirby et al., 2019	1	0, non-blinded	0, 11% loss to f/u	1, at the household level	0, caregiver recall	0, assessors/participants not blinded	2	**4**
Kirby et al., 2017	non-randomised study	1, negative control used	0, Data were missing for 12.6% of participants, and missing data disproportionately affected the intervention group, in which data was not available for 19.5% of participants.	1, at the household level	0, caregiver recall	0, assessors/participants not blinded	2	**4**

Peletz et al., 2012	1	0, non-blinded	0, 87% of data was available for intervention group and 81% of data was available for control group	1, at the household level	0, caregiver recall	0, assessor/participants not blinded	2	**4**
Boisson et al., 2010	1	1, participants blinded	0, 82.3% of total person-weeks of diarrhoea was analysed in the control group, 81.3% of total person-weeks of diarrhoea was analysed in control group	1, at the household level	0, caregiver recall	1, assessors/participants blinded	2	**6**
